# Right pulmonary to left pulmonary perfusion ratio after percutaneous pulmonary valve implantation

**DOI:** 10.1186/1532-429X-14-S1-P103

**Published:** 2012-02-01

**Authors:** Tobias Rutz, Manuel Seligmann, Christian Meierhofer, Heiko Stern, H Rieger, Petra Wolf, Stefan Martinoff, Andreas Eicken, John Hess, Sohrab Fratz

**Affiliations:** 1 Department of Paediatric Cardiology and Congenital Heart Disease, German Heart Centre, Munich, Germany

## Background

Percutaneous pulmonary valve implantation (PPVI) requires the positioning of a stiff guidewire into one of the pulmonary branch arteries. An infrequent but commonly known complication is the “jailing” of one of the pulmonary branch arteries. However, even without apparent “jailing” of one of the pulmonary branch arteries, we suspect that the technique of stent placements often has an effect on the pulmonary perfusion ratio.

The aim of this study was to assess the changes of the pulmonary perfusion ratio and their predictors after PPVI.

## Methods

We studied 47 consecutive patients who received PPVI without jailing of one the pulmonary branch arteries between December 2006 and September 2011. All patients underwent phase-velocity magnetic resonance before and after PPVI. Percent right pulmonary blood (RPA) flow was calculated as follows: percent right pulmonary blood flow = 100 x (RPA net flow volume / (RPA net flow volume + left pulmonary artery net flow volume)).

A change of more than +/- 10% in percent RPA flow was considered as a significant change in pulmonary perfusion ratio.

The positioning of the guide wire and the balloon during the intervention in respect to the protrusion into one of the pulmonary branch arteries was reviewed.

## Results

The pulmonary perfusion ratio changed in 17 % of patients significantly by 15 ± 4 %. The direction of the change of percent RPA flow was related in all these patients to the position of balloon during implantation of the most distal stent.

## Conclusions

PPVI without pulmonary branch intervention leads to a significant change in pulmonary perfusion ratio in 17% of patients. The change in pulmonary perfusion ratio is determined by the location of the balloon during implantation of the most distally situated stent. The clinical relevance of this new observation needs to be determined by prospective studies.

## Funding

No funding.

